# Manual of Physiology

**Published:** 1849-07

**Authors:** 


*Manual of Physiology*, by Wm. Senhouse Kirks, M. D., assisted by James Paget, Lecturer on General anatomy and Physiology at St. Bartholomew's Hospital; with one hundred and eighteen illustrations on wood. Philadelphia: Lea & Blanchard, 1849, 12 mo, pp. 548.

This is a reprint, by the enterprising American Publishers, Lea & Blanchard, without other alteration than substituting the term Manual for that of Hand-book, and interspersing the illustrations throughout the book, which are well executed in wood, instead of steel as in the original.

The unpretending volume before us seems admirably adapted to the purpose for which it was published, viz., a concise, but clear and full summary of the science of Physiology at the present time: embracing the cell theory and all the improvements resulting from the microscopical observations and chemical researches of the last few years. It was evidently not intended to supply the place, or supersede the more voluminous and elaborate works of Dunglison, Carpenter, Todd and Bowman, and others.

The older works on Physiology, have become almost useless from the new light thrown upon the subject by recent investigation, and what was wanted by the student, was a concise summary of all that is known, or admitted as established, without going into a history of the discovery, or a minute examination of the evidence upon which it rests.

For the general reader some account of the anatomy of the several organs, as given by Dunglison, would be desirable, but the student, for whom the work is particularly intended, is expected to have this knowledge, or to obtain it from another source.

In admitting as established the theory of Bischoff, Pouchet, Lee, and others, that the Graafian vesicles are developed and matured at regular intervals, corresponding with the menstrual period, independently of sexual connexion, our authors have gone further than any of the systematic works which have preceded them. So, also, in their account of the functions of the cerebral hemispheres, as being the instruments of the intellectual and moral faculties, and the admission that separate portions of the cerebrum perform separate and distinct functions, and that the power of the part, or organ, other things being equal, is in proportion to its size. They say that this does not constitute Phrenology. We are not disposed to cavil about the name, if the principle is admitted. We believe our authors have not gone further than they were justified in doing by the facts of science, or than would be sanctioned by the intelligent portion of the profession.

We conceive the work admirably adapted as a text book for students attending lectures on physiology, and would strongly recommend it as such.

